# Direct Assessment of Cumulative Aryl Hydrocarbon Receptor Agonist Activity in Sera from Experimentally Exposed Mice and Environmentally Exposed Humans

**DOI:** 10.1289/ehp.0901113

**Published:** 2009-12-14

**Authors:** Jennifer J. Schlezinger, Pamela L. Bernard, Amelia Haas, Philippe Grandjean, Pal Weihe, David H. Sherr

**Affiliations:** 1 Department of Environmental Health, Boston University School of Public Health, Boston, Massachusetts, USA; 2 Department of Medicine, Boston University School of Medicine, Boston, Massachusetts, USA; 3 Department of Environmental Medicine, University of Southern Denmark, Odense, Denmark; 4 Department of Environmental Health, Harvard School of Public Health, Boston, Massachusetts, USA; 5 Department of Occupational Medicine and Public Health, Faroese Hospital System, Torshavn, Faroe Islands

**Keywords:** aryl hydrocarbon receptor, dioxin toxicity, Faroe Islands, human serum, immunotoxicity, polychlorinated biphenyl

## Abstract

**Background:**

Aryl hydrocarbon receptor (AhR) ligands adversely affect many biological processes. However, assessment of the significance of human exposures is hampered by an incomplete understanding of how complex mixtures affect AhR activation/inactivation.

**Objectives:**

These studies used biological readouts to provide a broader context for estimating human risk than that obtained with serum extraction and gas chromatography/mass spectroscopy (GC/MS)-based assays alone.

**Methods:**

AhR agonist activity was quantified in sera from dioxin-treated mice, commercial human sources, and polychlorinated biphenyl (PCB)–exposed Faroe Islanders using an AhR-driven reporter cell line. To validate relationships between serum AhR agonist levels and biological outcomes, AhR agonist activity in mouse sera correlated with toxic end points. AhR agonist activity in unmanipulated (“neat”) human sera was compared with these biologically relevant doses and with GC/MS-assayed PCB levels.

**Results:**

Mouse serum AhR agonist activity correlated with injected dioxin dose, thymic atrophy, and heptomegaly, validating the use of neat serum to assess AhR agonist activity. AhR agonist activity in sera from Faroe Islanders varied widely, was associated with the frequency of recent pilot whale dinners, but did not correlate with levels of PCBs quantified by GC/MS. Surprisingly, significant “baseline” AhR activity was found in commercial human sera.

**Conclusions:**

An AhR reporter assay revealed cumulative levels of AhR activation potential in neat serum, whereas extraction may preclude detection of important non-dioxin-like biological activity. Significant levels of AhR agonist activity in commercial sera and in Faroe Islander sera, compared with that from experimentally exposed mice, suggest human exposures that are biologically relevant in both populations.

Research on polycyclic aromatic hydrocarbons (PAHs) and related halogenated aromatic hydrocarbons (HAHs) historically has focused on the ability of these common environmental contaminants to induce cell transformation. These contaminants also have been studied for their induction of a number of biological responses, including immunotoxicity. PAHs and HAHs reduce bone marrow and thymus cellularity, impair B- and T-lymphocyte proliferation, and alter B- and T-lymphocyte differentiation and function. The consequences of these alterations in immune system function include a decrease in pathogen and tumor immunity (Head and Lawrence 2009; Kerkvliet 2009) and potentially a skewing of immune responses toward pathologic autoimmunity (Funatake et al. 2005; Quintana et al. 2008; Veldhoen et al. 2008). Many PAHs and HAHs bind to and activate the aryl hydrocarbon receptor (AhR), and their toxic effects require this activation (Fernandez-Salguero et al. 1996). The AhR activates transcription of multiple genes, such as those responsible for metabolism of PAHs and HAHs [e.g., cytochrome P450 (CYP) genes], and suppresses the transcription of others (e.g., immunoglobulin) (Denison et al. 2002; Sulentic et al. 1998).

Hazard and risk assessment typically is determined for individual chemicals; however, human and wildlife exposures to environmental contaminants usually occur as mixtures. Human exposure to toxic AhR agonists frequently is determined by measuring, in serum extracts, individual common polychlorinated biphenyl (PCB) or polychlorinated dibenzo-*p*-dioxin/furan (PCDD/PCDF) congeners by high-resolution gas chromatography/mass spectrometry (GC/MS) and then calculating a total “toxic equivalence” (TEQ) based on the ability of each compound to induce toxic effects (the sum of the products of each compound’s concentration multiplied by its TEQ value) (Van den Berg et al. 2006). Two critical assumptions of this approach may influence the risk assessment of exposure to AhR agonist mixtures. This method does not account for *a*) the antagonistic behavior of *ortho*-substituted PCBs (Harper et al. 1995; Howard et al. 2007) or *b*) the agonistic or antagonistic behavior and toxicity of nonchlorinated AhR ligands (Denison and Nagy 2003) (e.g., PAHs, indoles, and flavonoids), all of which occur at much higher concentrations in the environment than do dioxin-like compounds (Safe 1998) and would be excluded by serum extraction.

One advance in the assessment of AhR ligand mixture exposure has been the use of AhR-dependent bioassays, such as the chemical-activated luciferase gene expression (CALUX) assay (Windal et al. 2005). The attraction of this approach lies in its potential to assess the cumulative biological effects of a mixture of extractable AhR ligands, whether the effect of an individual ligand is agonistic or antagonistic. Some studies have demonstrated a strong association between AhR activity, as measured in acid silica-column–processed serum extracts by CALUX, and TEQ calculated from GC/MS assessment (*r* > 0.7) (Covaci et al. 2002; Pauwels et al. 2000; Van Den Heuvel et al. 2002). However, other studies report finding little or no correlation (*r* < 0.6), with a population-dependent range of correlations determined in a single study (*r* = 0.43–0.73) (Koppen et al. 2001; Porpora et al. 2009; Van Wouwe et al. 2004; Warner et al. 2005). These differences could reflect the biological interaction of multiple full AhR agonists, partial agonists, and antagonists quantified in the CALUX assay but overlooked in the GC/MS assay.

Given the role that the AhR plays in critical physiologic functions and the potential limitations in assessing the significance of exposures to contaminant mixtures, we propose that a broader perspective and a complementary biological approach be considered, specifically because the cumulative biological effects of agonists, partial agonists, and antagonists are impossible to predict from analytical assays. We hypothesize that adaptation of CALUX-like methods to use with whole, unmanipulated (“neat”) sera (Ziccardi et al. 2000) would avoid exclusion of some AhR agonists/antagonists by extraction of sera and would provide biologically relevant insights into the significance of human exposure to complex mixtures of AhR agonists and antagonists.

## Materials and Methods

### Animals and treatment

Animal studies were conducted in accordance with the Boston University Institutional Animal Care and Use Committee guidelines for humane treatment of animals and alleviation of suffering. Female 5- to 7-week-old C57BL/6J mice were purchased from Jackson Laboratory (Bar Harbor, ME, USA). Six to 18 mice were dosed by intraperitoneal injection with vehicle (sesame oil) or 2,3,7,8-tetrachlorodibenzo-*p*-dioxin (TCDD; 2.5, 5, 10, or 30 μg/kg; Ultra Scientific, North Kingstown, RI, USA). Eight days after treatment, blood was collected and pooled from two mice for each sample. The mice were euthanized, and the body weights were measured. Thymi and livers were resected and weighed. The number of viable thymocytes was determined by trypan blue staining and counting using a hemocytometer.

### Human sera

Approval of the protocol was obtained from the Faroese Ethical Review Committee and the Institutional Review Board at Harvard School of Public Health. Human subjects gave written informed consent before the study. Blood samples were collected and sera prepared from pregnant women (gestational week 32) in 1994–1995 (cohort 2) and in 2000–2001 (cohort 3) in the Faroe Islands. Dietary information was available from 149 and 211 women in each cohort, respectively. Serum samples were analyzed for the major PCB congeners by GC with electron-capture detection (Petersen et al. 2006). The sum of PCBs was calculated as the sum of PCB-138, PCB-153, and PCB-180 multiplied by 2 (Grandjean et al. 1995). The estimated dioxin-like activity of the major mono-*ortho*-PCBs was computed as the sum of PCB-105, PCB-118, and PCB-156, where the latter was weighted 5-fold higher according to the World Health Organization TEQs (Van den Berg et al. 1998). The sum of PCBs and the estimated dioxin-like activity were highly correlated (*r* = 0.95).

All other human AB sera were purchased from commercial sources: SeraCare (Oceanside, CA, USA), ICN Biomedicals (Solon, OH, USA), Sigma (St. Louis, MO, USA), and Dynal (Invitrogen, Carlsbad, CA, USA). These sera were all collected in the United States.

### Serum preparation

Serum was prepared by allowing the blood to clot and then removing the clot. Serum extractions and cleanup were performed as described previously (Murk et al. 1997) [see Supplemental Material (doi:10.1289/ehp.0901113)]. Activated charcoal was used to remove non-*ortho*-substituted PCBs (Jensen and Sundtrom 1974) (see Supplemental Material). This method was validated to remove all AhR agonist activity in serum supplemented with 10^−4^ M Aroclor 1242 (see Supplemental Material, Figure 1).

### Assessment of AhR activity

H1G1.1c3 cells were generously provided by M.S. Denison (University of California, Davis, CA, USA). Cells were maintained and prepared for experiments as described previously (Nagy et al. 2002), except that cells were plated at a concentration of 7.5 × 10^4^ cells per well and sera were applied in 10-μL aliquots [see Supplemental Material (doi:10.1289/ehp.0901113)]. In specificity experiments, wells were pretreated with either DMSO (0.5% final concentration) or the AhR antagonist CH223191 (10−^5^ M final concentration) (EMD Chemicals, San Diego, CA, USA). For calculation of serum AhR agonist activity, the standard curve was fitted using the four-parameter sigmoid Hill equation. Triplicate fluorescence measurements for each serum sample were averaged and used to determine the serum AhR agonist activity, in picograms TCDD equivalents (eq) per milliliter, by interpolation from the fitted standard curve (see Supplemental Material, Figure 2).

### Statistics

Data are reported as means ± SE. Linear and nonlinear (exponential) regression analyses (Sigma Plot; Systat Software, Chicago, IL, USA) were used where appropriate to determine *r* and *r*^2^. Statistical analyses were performed with Statview (SAS Institute Inc., Cary, NC, USA). Student’s *t*-tests, the *t*-statistic, and one-factor analyses of variance (ANOVAs) combined with Dunnett’s test or the Tukey–Kramer multiple comparisons test were used to determine significance.

## Results

### Validation and calibration of a neat serum AhR agonist activity assay in a mouse model of TCDD toxicity

Exposure to AhR agonists results in receptor-dependent immunotoxic and hepatotoxic effects in animal models (Fernandez-Salguero et al. 1996). To validate the use of neat serum in a sensitive reporter assay for AhR activation and to calibrate the injected dose of TCDD with serum AhR agonist activity and toxic end points *in vivo*, C57BL/6 mice were dosed with vehicle or TCDD by intraperitoneal injection.

**Figure 1:**
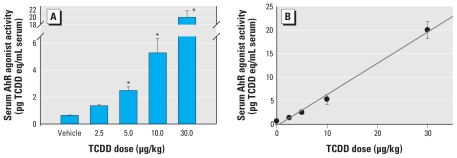
TCDD injection results in biologically active serum TCDD concentrations, as determined in neat serum applied to H1G1.1c3 cells containing an AhR reporter. (*A*) Means ± SE from three to nine pools of sera. (*B*) Linear regression: *r*^2^ = 0.952; *r* = 0.976; *p* < 0.01. *Significantly different from control, *p* < 0.05 (ANOVA, Dunnett’s test).

Mouse serum was applied directly to mouse hepatoma cells stably transfected with an AhR-driven enhanced green fluorescent protein (EGFP)-expressing reporter construct with a promoter similar to that in the AhR–CALUX assay (Nagy et al. 2002). These assays have been shown to produce similar results, with the advantage that the EGFP provides a nondestructive readout (Nagy et al. 2002; Nording et al. 2007; Peters et al. 2006). We calculated AhR agonist activity values, presented as picograms TCDD equivalents (eq) per milliliter, by interpolation from TCDD standard curves. Although sometimes referred to as TEQs, this measure itself is not a measure of toxicity. We observed a significant increase in AhR agonist activity after injection of ≥ 5 μg/kg TCDD (Figure 1A). The increase was linear and highly correlated with the injected TCDD dose (Figure 1B). Although expected, these results rigorously demonstrate for the first time a linear relationship between AhR agonist exposure and TCDD equivalents measured in neat sera.

**Figure 2:**
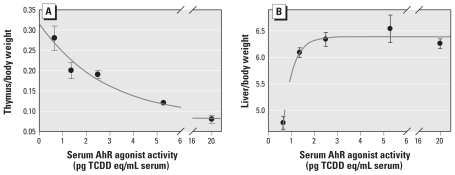
TCDD treatment results in thymic atrophy (*A*) and hepatomegaly (*B*) that correlate with serum AhR agonist activity. Data are means ± SE from three to nine data points from organs grouped by serum pooling. Nonlinear regressions between serum AhR agonist activity and toxic end points: *A*, *r*^2^ = 0.964; *r* = 0.979; *B*, *r*^2^ = 0.980; *r* = 0.990.

The thymus is an extremely sensitive TCDD target, with exposure resulting in AhR-dependent thymic involution (Staples et al. 1998). We observed statistically significant decreases in thymus:body weight ratios and total thymocyte number at a dose as low as 2.5 μg TCDD/kg [see Supplemental Material, Figure 3 (doi:10.1289/ehp.0901113)]. Thymus:body weight ratios correlated inversely but nonlinearly with mouse serum AhR agonist activity (Figure 2A). Similarly, liver:body weight ratios increased with dose of TCDD (see Supplemental Material, Figure 4) and correlated with mouse serum AhR agonist activity (Figure 2B). These *in vivo* results strongly support the hypothesis that AhR agonist activity, determined in neat serum samples, correlates with injected TCDD dose and toxicity.

### Analysis of AhR agonist activity in neat sera from human populations

As part of an ongoing study on the effects of environmental contaminants on the immune system, blood samples were collected from two cohorts of pregnant women from the Faroe Islands. We evaluated sera from 156 cohort 2 donors (1994–1995) and from 227 cohort 3 donors (2000–2001). In this community, the traditional seafood diet includes pilot whale, the blubber of which contains elevated concentrations of persistent organic pollutants (Dam and Bloch 2000), which results in elevated exposures to PCBs, but not to PCDD/PCDFs (Grandjean et al. 1995; Heilmann et al. 2006). Based on toxicologic evidence, the Faroese health authorities in 1998 recommended that women of reproductive age should avoid eating whale.

**Figure 3:**
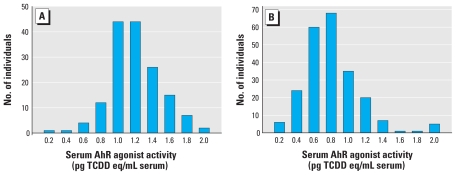
Distribution of serum AhR agonist activity measured in two cohorts of a Faroe Island population environmentally exposed to PCBs: cohort 2 (1994–1995; *A*) and cohort 3 (2000–2001; *B*). On the *x*-axis, the serum AhR agonist activity shown indicates the maximum of the range for each group of individuals.

For assessment of AhR agonist activity, we applied human sera to H1G1.1c3 cells as described for the mouse studies. Results from cohort 2 indicated a range of serum AhR agonist activity, with 24 samples (90th percentile) exhibiting ≥ 1.4 pg TCDD eq/mL (Figure 3A), the serum AhR agonist activity measured in mouse sera from TCDD-treated mice exhibiting thymic and hepatic toxicity. Interestingly, the average AhR agonist activity in sera from cohort 2 (1.09 ± 0.02 pg TCDD eq/mL) was significantly higher than the AhR agonist activity in sera from vehicle-treated mice (e.g., Figure 1; 0.65 ± 0.04 pg TCDD eq/mL; *p* < 0.01, Student’s *t*-test). Furthermore, we observed a significantly lower average level of AhR agonist activity in the sera of cohort 3 donors (Figure 3B) than in cohort 2 donors (1.09 ± 0.02 vs. 0.71 ± 0.02 pg TCDD eq/mL; *p* < 0.01, Student’s *t*-test), with only individuals in the 97th percentile exhibiting serum AhR agonist activities ≥ 1.4 pg TCDD eq/mL, suggesting a decrease in exposure to AhR agonists during the interval between collection of samples from each cohort and after issuance of a health advisory concerning whale consumption.

In cohort 2, 54 women had no whale meals during pregnancy, 41 and 25 had one and two whale meals per month, respectively, and 29 were more frequent consumers. The AhR agonist activity did not differ among the first three groups and showed an overall average of 1.07 ± 0.03 pg TCDD eq/mL, whereas the average for high consumers was 1.18 ± 0.06 pg TCDD eq/mL. In cohort 3, whale consumption was much less frequent. A total of 75 women had eaten no whale, 87 had up to three whale meals during the pregnancy, and 49 had eaten whale more frequently and up to once per week. The AhR agonist activity did not differ between the first two groups and showed an overall average of 0.69 ± 0.02 pg TCDD eq/mL, whereas the average for high consumers was 0.80 ± 0.07 pg TCDD eq/mL (*p* = 0.07, *r* = 0.13). If the nonconsumers were omitted, the correlation improved (*r* = 0.17, *p* = 0.04). For the combined data from both cohorts, the correlation (*r* = 0.27) was highly significant (*p* < 0.001).

**Figure 4:**
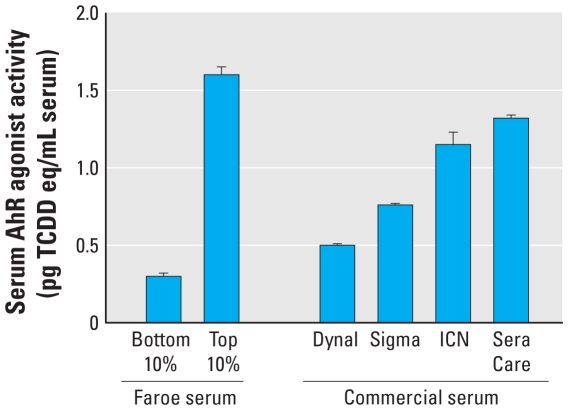
Comparison between serum AhR agonist activity in Faroe Islands cohorts and that in commercial human serum: means ± SE of 10 individual sera for the Faroe Island samples and three separate aliquots for the commercial sera.

Because of this considerable range in serum AhR activity within and between the two Faroe Islands cohorts, and the apparently lower level of baseline AhR agonist activity in mouse compared with human sera, we examined the levels of “baseline” serum AhR agonist activity in sera from the general U.S. population. We determined AhR agonist activity in pools of human serum samples, each from one of four independent commercial sources, and compared these with individual sera from Faroe Islanders previously shown to express the highest (upper 10%, 1.60 ± 0.05 pg TCDD eq/mL serum) or lowest (lower 10%, 0.30 ± 0.02 pg TCDD eq/mL serum) serum AhR agonist activity (e.g., Figure 3). Surprisingly, the four pools of commercial sera differed in their level of AhR agonist activity, varying from a low of 0.50 ± 0.01 pg TCDD eq/mL serum (Dynal) to a high of 1.32 ± 0.02 pg TCDD eq/mL serum (SeraCare) (Figure 4). These results suggest that significant levels of AhR agonist activity in human populations are not uncommon. It is unknown whether this background AhR agonist activity results from exposure to exogenous AhR ligands or to production of endogenous ligands.

Having quantified the cumulative level of AhR agonist activity in sera from Faroe Islanders, we next evaluated whether the biological readout correlated with PCB exposure. Although elevated levels of PCBs have been found in this population (Grandjean et al. 1995), we observed no significant relationship between summed PCB levels and Faroe serum AhR agonist activity determined in the bioassay [see Supplemental Material, Figure 5A (doi:10.1289/ehp.0901113)]. To determine whether serum extraction potentially influenced the AhR agonist activity measured in the typical CALUX-like assay, we prepared serum extracts and compared the AhR agonist activities in the Faroe serum extracts and in neat sera. Although we observed a slight upward trend in the comparison, we found minimal correlation between AhR agonist activity in the extract and in the neat Faroe sera (see Supplemental Material, Figure 5B). These data suggest that extraction and processing procedures may alter the relative composition of AhR agonists and/or antagonists in sera, thereby influencing either a chemical or biological assessment of toxicity.

**Figure 5:**
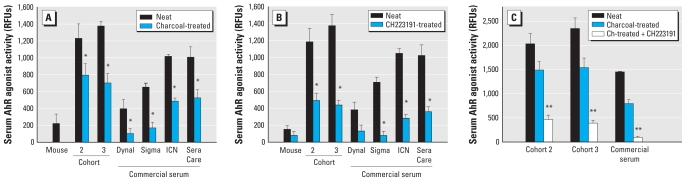
Charcoal stripping or AhR antagonist cotreatment significantly reduces AhR activity induced by human serum: comparison of AhR agonist activity in neat and charcoal (ch)-treated sera (*A*), neat sera cotreated with vehicle or CH223191 (*B*), and charcoal-treated sera cotreated with vehicle or CH223191 (*C*; commercial serum = ICN, Sigma, and SeraCare sera). Ch-treated, charcoal-treated. Data are presented as means ± SE of seven individual sera for the Faroe Island samples and three aliquots of each of the commercial sera. *Significantly different from neat serum, *p* < 0.05 (Student’s *t*-test). **Significantly different from charcoal treated, *p* < 0.05 (ANOVA, Tukey–Kramer test).

We examined the contribution of planar HAHs to the observed AhR agonist activity by depletion of these AhR agonists from sera by charcoal stripping (Jensen and Sundtrom 1974). Samples from both Faroe Islands cohorts exhibiting the highest (10%) serum AhR agonist activity and the commercial human serum pools were treated with activated charcoal before assessment of serum AhR agonist activity. Charcoal treatment resulted in a significant, although incomplete, reduction in serum AhR agonist activity (Figure 5A). Next, we determined the effect of a pure AhR antagonist, CH223191 (Kim et al. 2006), on the ability of sera to induce reporter activity. H1G1.1c3 cells were pretreated either with vehicle or with CH223191 before application of sera. CH223191 inhibited serum AhR agonist activity in samples from cohort 2, cohort 3, and the four commercial samples, although inhibition of activity in the Dynal serum failed to reach statistical significance (Figure 5B). Interestingly, CH223191 appeared to reduce the serum AhR agonist activity measured in human sera to a greater extent than did charcoal treatment, and CH223191 significantly reduced the AhR agonist activity measured in charcoal-treated serum (Figure 5C), confirming that the charcoal-resistant activity was AhR specific. Thus, we conclude that a significant fraction of AhR agonist activity found in both an HAH-exposed and the general U.S. population is not removed by a standard procedure known to remove dioxin-like AhR ligands.

## Discussion

Assessment of human exposure to toxic AhR ligands traditionally relies on GC/MS analysis of common PCB and PCDD/PCDF congeners extracted from serum. Although this method can specifically identify and quantify HAH congeners, estimating the toxicity of the exposure relies on extrapolation to the biological AhR-activating capacity of the mixture using the TEQ method (Van den Berg et al. 2006). Furthermore, the estimation of toxicity from a calculated TEQ relies on several assumptions: that the effect of all AhR ligands is positively additive, that all biologically relevant chemicals are equally extractable from samples, and that AhR activation, regardless of the inducing agent, is a monolithic quantitative event that always results in an outcome equivalent to that seen after activation with TCDD. Here, we demonstrate that caution should be taken with regard to at least some of these assumptions.

The central hypothesis driving these studies was that analysis of AhR agonist activity in neat serum, as a cumulative measure of AhR ligand (agonist, partial agonist, antagonist) exposure, would provide a broader context for interpretation of GC/MS-determined TEQs and exposure assessment. To begin to test the hypothesis, we determined the relationship between controlled TCDD exposures and serum AhR agonist activity in a mouse model. Mouse serum AhR agonist activity linearly correlated with the injected TCDD dose, validating the use of neat sera to detect circulating AhR agonists.

We then extended studies of AhR activity to analyses of sera from a human population exposed to HAHs in the diet and analyses of commercially available human sera pools. Several characteristics of the data were of note. First, a range of serum AhR activity was measured in samples from the Faroe Islands population, suggesting that either pharmacokinetics or exposures to AhR agonists may differ within this population. Interestingly, the distribution of serum AhR agonist activity was higher in cohort 2 than in the more recent cohort 3, samples from which were collected after a dietary advisory to abstain from eating whale. Because the demographics of the participants in these two cohorts were similar, this decrease, and the association with the frequency of whale dinners during pregnancy, may reflect a decrease in whale blubber consumption. Additionally, this decrease may reflect a significant decrease in HAH emissions and deposition over this time (Hays and Aylward 2003).

Second, “baseline” serum AhR agonist activity in the commercial serum pools, collected from the U.S. population at large, differed considerably among sources for each serum pool. Additionally, two of the commercial human sera (ICN and SeraCare) had significantly greater serum AhR agonist activity than did sera from vehicle-treated mice, and their activity falls within the 70th and 90th percentile of Faroe cohorts 2 and 3, respectively. Given the grain-based feed and short life span of the mice (i.e., limited AhR agonist exposure), it is tempting to speculate that the relatively high AhR agonist activity in some of the commercial sera is atributable to accumulated and ongoing exposures to environmental AhR ligand pollutants. Alternatively, exposure to PAHs and natural ligands found in plants could contribute to the total AhR activity measured in neat human serum. Recent studies have shown that the AhR-activating capacity of extracted, but unprocessed, serum could be influenced significantly by consumption of a vegetable-based diet in humans (Connor et al. 2008; de Waard et al. 2008). For the Faroe Islanders, high AhR agonist activity could reflect exposure to natural AhR ligands found in whale blubber (Vetter et al. 2005). Also, endogenous, serum-borne ligands, such as bilirubin, biliverdin, and tryptophan metabolites (Denison and Nagy 2003), exhibit AhR agonist activity and could contribute to the high level of serum AhR activity in humans.

Third, AhR agonist activity measured in Faroe serum was not significantly correlated with summed PCB exposure and was partially resistant to charcoal treatment. Taken together, the data suggest that a significant portion of AhR agonist activity in the Faroe serum is not mediated by charcoal-absorbable, dioxin-like, non-*ortho*-PCB congeners. Interestingly, comparison of PCB exposure and activity of CYP1A2, an enzyme whose expression is controlled by AhR, in the Faroe Islands population also did not reveal a correlation (Petersen et al. 2006). Previous studies have demonstrated that, whereas the CALUX assay–determined and the GC/MS-determined TEQs are highly correlated when only the PCDD/PCDF fraction of serum is analyzed, the correlation dissipates when the PCB fraction is included (Van Wouwe et al. 2004; Windal et al. 2005). Thus, the CALUX assay provides an accurate assessment of PCDD/PCDF exposure only when the PCDD/PCDF fraction alone is used. This also is illustrated by the results shown here, with a highly significant correlation between mouse serum AhR activity and TCDD dose.

When the GC/MS-determined TEQs do not correspond to CALUX-determined AhR activities, the question then becomes, what added information is the CALUX assay indicating about toxic potency? We demonstrate that mouse serum AhR activity correlates, in an asymptotic manner, with toxic end points (thymic atrophy and hepatomegaly) characteristic of TCDD exposure. However, exposures tend to be complex, containing both agonistic and antagonistic components. Analysis of PCDD/PCDF congeners alone would not take into account the potentially ameliorating effects of antagonistic compounds. AhR antagonists found in the diet or synthesized endogenously could negatively affect AhR activation. Natural products, including flavonoids, catechins, curcumin, resveratrol, and lutein, all antagonize the AhR (Denison and Nagy 2003). In addition, Aroclors and *ortho*-substituted PCBs contain or are partial agonists that suppress TCDD-induced immunotoxicity, presumably through competitive binding to the AhR (Peters et al. 2006; Suh et al. 2003).

Finally, it has been argued that only chemicals that are dioxin-like in structure and lead to AhR-dependent acute toxicity (as opposed to CYP-mediated toxicity of PAHs) should be considered in toxic potency analyses (Van den Berg et al. 2006). Considering that as much as 98% of the total daily AhR agonist intake could be attributable to non-dioxin-like compounds (Safe 1998), we suggest that AhR-dependent toxicity should not be defined by this conventional syndrome alone. Although some AhR agonists, such as PAHs, do not persist in biological systems like TCDD, they are highly toxic to a number of organ systems, including the immune (Mann et al. 1999), endocrine (Matikainen et al. 2002), and neurologic systems (McCallister et al. 2008). Using the immune system as a model for non-dioxin-like, AhR-mediated toxicity, recent studies demonstrated that AhR-activating tryptophan metabolites influence development of T-cell subsets that control inflammation, autoimmunity, and tumor immunity (Quintana et al. 2008; Veldhoen et al. 2008). Thus, consideration of the potential for non-dioxin-like AhR agonists to alter many physiologic functions (i.e., “toxicity”) is warranted.

## Conclusions

These studies demonstrate that a sensitive reporter assay can be adapted to detect the cumulative AhR agonist activity in neat mammalian sera. Within limits (e.g., considering species differences in AhR affinity), the mouse model provides a system with which to relate serum AhR agonist activities with toxicologic end points and could be used to investigate the relationship between these factors in mixture exposures. The human studies here and elsewhere demonstrate the need for an assay that measures cumulative AhR activity without the need to know a priori the likely composition of and interactions between AhR agonist and antagonist mixtures and without a possible bias imposed by evaluation of only extractable dioxin-like compounds. These results highlight the need to perform both standard chemical analyses and bioassays to evaluate human exposure to environmental AhR ligands and ultimately to estimate risk.

## References

[b1-ehp-118-693] Connor KT, Harris MA, Edwards MR, Budinsky RA, Clark GC, Chu AC (2008). AH receptor agonist activity in human blood measured with a cell-based bioassay: evidence for naturally occurring AH receptor ligands *in vivo*. J Expo Sci Environ Epidemiol.

[b2-ehp-118-693] Covaci A, Koppen G, Van Cleuvenbergen R, Schepens P, Winneke G, van Larebeke N (2002). Persistent organochlorine pollutants in human serum of 50–65 years old women in the Flanders Environmental and Health Study (FLEHS). Part 2: correlations among PCBs, PCDD/PCDFs and the use of predictive markers. Chemosphere.

[b3-ehp-118-693] Dam M, Bloch D (2000). Screening of mercury and persistent organochlorine pollutants in long-finned pilot whale (*Globicephala melas*) in the Faroe Islands. Mar Pollut Bull.

[b4-ehp-118-693] de Waard PW, Peijnenburg AA, Baykus H, Aarts JM, Hoogenboom RL, van Schooten FJ (2008). A human intervention study with foods containing natural Ah-receptor agonists does not significantly show AhR-mediated effects as measured in blood cells and urine. Chem Biol Interact.

[b5-ehp-118-693] Denison MS, Nagy SR (2003). Activation of the aryl hydrocarbon receptor by structurally diverse exogenous and endogenous chemicals. Annu Rev Pharmacol Toxicol.

[b6-ehp-118-693] Denison MS, Pandini A, Nagy SR, Baldwin EP, Bonati L (2002). Ligand binding and activation of the Ah receptor. Chem Biol Interact.

[b7-ehp-118-693] Fernandez-Salguero PM, Hilbert DM, Rudikoff S, Ward JM, Gonzalez F (1996). Aryl hydrocarbon receptor-deficient mice are resistant to 2,3,7,8-tetrachlorodibenzo-*p*-dioxin-induced toxicity. Toxicol Appl Pharmacol.

[b8-ehp-118-693] Funatake CJ, Marshall NB, Steppan LB, Mourich DV, Kerkvliet NI (2005). Cutting edge: activation of the aryl hydrocarbon receptor by 2,3,7,8-tetrachlorodibenzo-*p*-dioxin generates a population of CD4+ CD25+ cells with characteristics of regulatory T cells. J Immunol.

[b9-ehp-118-693] Grandjean P, Weihe P, Needham LL, Burse VW, Patterson DG, Sampson EJ (1995). Relation of a seafood diet to mercury, selenium, arsenic, and polychlorinated biphenyl and other organochlorine concentrations in human milk. Environ Res.

[b10-ehp-118-693] Harper N, Connor K, Steinberg M, Safe S (1995). Immunosuppressive activity of polychlorinated biphenyl mixtures and congeners: nonadditive (antagonistic) interactions. Fundam Appl Toxicol.

[b11-ehp-118-693] Hays SM, Aylward LL (2003). Dioxin risks in perspective: past, present, and future. Regul Toxicol Pharmacol.

[b12-ehp-118-693] Head JL, Lawrence BP (2009). The aryl hydrocarbon receptor is a modulator of anti-viral immunity. Biochem Pharmacol.

[b13-ehp-118-693] Heilmann C, Grandjean P, Weihe P, Nielsen F, Budtz-J⊘rgensen E (2006). Reduced antibody responses to vaccinations in children exposed to polychlorinated biphenyls. PLoS Med.

[b14-ehp-118-693] Howard JG, Schlezinger JJ, Webster TF (2007). Interactions of TCDD with AhR partial agonists and a competitive antagonist: implications for TEFs. Organohalogen Compounds.

[b15-ehp-118-693] Jensen S, Sundtrom G (1974). Structures and levels of most chlorobiphenyls in two technical PCB products. Ambio.

[b16-ehp-118-693] Kerkvliet NI (2009). AHR-mediated immunomodulation: the role of altered gene transcription. Biochem Pharmacol.

[b17-ehp-118-693] Kim SH, Henry EC, Kim DK, Kim YH, Shin KJ, Han MS (2006). Novel compound 2-methyl-2H-pyrazole-3-carboxylic acid (2-methyl-4-o-tolylazo-phenyl)-amide (CH-223191) prevents 2,3,7,8-TCDD-induced toxicity by antagonizing the aryl hydrocarbon receptor. Mol Pharmacol.

[b18-ehp-118-693] Koppen G, Covaci A, Van Cleuvenbergen R, Schepens P, Winneke G, Nelen V (2001). Comparison of CALUX-TEQ values with PCB and PCDD/F measurements in human serum of the Flanders Environmental and Health Study (FLEHS). Toxicol Lett.

[b19-ehp-118-693] Mann K, Matulka R, Hahn M, Trombino A, Lawrence B, Kerkvliet N (1999). The role of polycyclic aromatic hydrocarbon metabolism in dimethylbenz[*a*]anthracene-induced pre-B lymphocyte apoptosis. Toxicol Appl Pharmacol.

[b20-ehp-118-693] Matikainen TM, Moriyama T, Morita Y, Perez GI, Korsmeyer SJ, Sherr DH (2002). Ligand activation of the aromatic hydrocarbon receptor transcription factor drives Bax-dependent apoptosis in developing fetal ovarian germ cells. Endocrinology.

[b21-ehp-118-693] McCallister MM, Maguire M, Ramesh A, Aimin Q, Liu S, Khoshbouei H (2008). Prenatal exposure to benzo(a)pyrene impairs later-life cortical neuronal function. Neurotoxicology.

[b22-ehp-118-693] Murk A, Leonards P, Bulder A, Jonas A, Roxemeijer M, Denison M (1997). The CALUX (chemical-activated luciferase expression) assay adapted and validated for measuring TCDD equivalents in blood plasma. Environ Toxicol Chem.

[b23-ehp-118-693] Nagy SR, Sanborn JR, Hammock BD, Denison MS (2002). Development of a green fluorescent protein-based cell bioassay for the rapid and inexpensive detection and characterization of Ah receptor agonists. Toxicol Sci.

[b24-ehp-118-693] Nording M, Denison MS, Baston D, Persson Y, Spinnel E, Haglund P (2007). Analysis of dioxins in contaminated soils with the CALUX and CAFLUX bioassays, an immunoassay, and gas chromatography/high-resolution mass spectrometry. Environ Toxicol Chem.

[b25-ehp-118-693] Pauwels A, Cenijn PH, Schepens PJ, Brouwer A (2000). Comparison of chemical-activated luciferase gene expression bioassay and gas chromatography for PCB determination in human serum and follicular fluid. Environ Health Perspect.

[b26-ehp-118-693] Peters AK, Leonards PE, Zhao B, Bergman A, Denison MS, Van den Berg M (2006). Determination of *in vitro* relative potency (REP) values for mono-*ortho* polychlorinated biphenyls after purification with active charcoal. Toxicol Lett.

[b27-ehp-118-693] Petersen MS, Halling J, Damkier P, Nielsen F, Grandjean P, Weihe P (2006). Caffeine N3-demethylation (CYP1A2) in a population with an increased exposure to polychlorinated biphenyls. Eur J Clin Pharmacol.

[b28-ehp-118-693] Porpora MG, Medda E, Abballe A, Bolli S, De Angelis I, di Domenico A (2009). Endometriosis and organochlorinated environmental pollutants: a case–control study on Italian women of reproductive age. Environ Health Perspect.

[b29-ehp-118-693] Quintana FJ, Basso AS, Iglesias AH, Korn T, Farez MF, Bettelli E (2008). Control of T(reg) and T(H)17 cell differentiation by the aryl hydrocarbon receptor. Nature.

[b30-ehp-118-693] Safe SH (1998). Development, validation and problems with the toxic equivalency factor approach for risk assessment of dioxins and related compounds. J Anim Sci.

[b31-ehp-118-693] Staples JE, Murante FG, Fiore NC, Gasiewicz TA, Silverstone AE (1998). Thymic alterations induced by 2,3,7,8-tetrachlorodibenzo-*p*-dioxin are strictly dependent on aryl hydrocarbon receptor activation in hemopoietic cells. J Immunol.

[b32-ehp-118-693] Suh J, Kang JS, Yang KH, Kaminski NE (2003). Antagonism of aryl hydrocarbon receptor-dependent induction of CYP1A1 and inhibition of IgM expression by di-*ortho*-substituted polychlorinated biphenyls. Toxicol Appl Pharmacol.

[b33-ehp-118-693] Sulentic CE, Holsapple MP, Kaminski NE (1998). Aryl hydrocarbon receptor-dependent suppression by 2,3,7,8-tetrachlorodibenzo-*p*-dioxin of IgM secretion in activated B cells. Mol Pharmacol.

[b34-ehp-118-693] Van den Berg M, Birnbaum L, Bosveld AT, Brunstrom B, Cook P, Feeley M (1998). Toxic equivalency factors (TEFs) for PCBs, PCDDs, PCDFs for humans and wildlife. Environ Health Perspect.

[b35-ehp-118-693] Van den Berg M, Birnbaum LS, Denison M, De Vito M, Farland W, Feeley M (2006). The 2005 World Health Organization reevaluation of human and mammalian toxic equivalency factors for dioxins and dioxin-like compounds. Toxicol Sci.

[b36-ehp-118-693] Van Den Heuvel RL, Koppen G, Staessen JA, Hond ED, Verheyen G, Nawrot TS (2002). Immunologic biomarkers in relation to exposure markers of PCBs and dioxins in Flemish adolescents (Belgium). Environ Health Perspect.

[b37-ehp-118-693] Van Wouwe N, Windal I, Vanderperren H, Eppe G, Xhrouet C, De Pauw E (2004). Importance of clean-up for comparison of TEQ-values obtained by CALUX and chemo-analysis. Talanta.

[b38-ehp-118-693] Veldhoen M, Hirota K, Westendorf AM, Buer J, Dumoutier L, Renauld JC (2008). The aryl hydrocarbon receptor links TH17-cell-mediated autoimmunity to environmental toxins. Nature.

[b39-ehp-118-693] Vetter W, Hahn ME, Tomy G, Ruppe S, Vatter S, Chahbane N (2005). Biological activity and physicochemical parameters of marine halogenated natural products 2,3,3′,4,4′,5,5′-heptachloro-1′-methyl-1,2′-bipyrrole and 2,4,6-tribromoanisole. Arch Environ Contam Toxicol.

[b40-ehp-118-693] Warner M, Eskenazi B, Patterson DG, Clark G, Turner WE, Bonsignore L (2005). Dioxin-Like TEQ of women from the Seveso, Italy area by ID-HRGC/HRMS and CALUX. J Expo Anal Environ Epidemiol.

[b41-ehp-118-693] Windal I, Denison MS, Birnbaum LS, Van Wouwe N, Baeyens W, Goeyens L (2005). Chemically activated luciferase gene expression (CALUX) cell bioassay analysis for the estimation of dioxin-like activity: critical parameters of the CALUX procedure that impact assay results. Environ Sci Technol.

[b42-ehp-118-693] Ziccardi M, Gardner I, Denison M (2000). Development and modification of a recombinant cell bioassay to directly detect halogenated and polycyclic aromatic hydrocarbons in serum. Toxicol Sci.

